# Exogenous proline promotes serum killing of *Klebsiella pneumoniae*

**DOI:** 10.1080/21505594.2025.2545558

**Published:** 2025-08-07

**Authors:** Tian-Shun Kou, Yan-Yan Shang, Qi-Chao Zhang, Si-Qi Tian, Juan Li, Li-Na Yang, Ling Min, Bo Peng

**Affiliations:** a Guangzhou Institute of Cancer Research, The Affiliated Cancer Hospital, Guangzhou Medical University, Guangzhou, China; b State Key Laboratory of Bio-Control, Guangdong Key Laboratory of Pharmaceutical Functional Genes, School of Life Sciences, Southern Marine Science and Engineering Guangdong Laboratory (Zhuhai), Sun Yat-Sen University, Guangzhou, China; c Laboratory for Marine Biology and Biotechnology, Qingdao Marine Science and Technology Center, Qingdao, China

**Keywords:** serum resistance, proline, *Klebsiella pneumoniae*, complement binding, pyruvate cycle

## Abstract

*Klebsiella pneumoniae*, a common pathogen responsible for bloodstream infections, can evade clearance by the complement-dependent killing in serum, known as serum resistance. However, strategy in managing *K. pneumoniae* serum resistance is still lacking. In this study, we employed metabolomics to identify the metabolic features of *K. pneumoniae*. We found that the pyruvate/TCA cycle and alanine, aspartate, and glutamate metabolic pathways were significantly downregulated. Proline, identified as a key biomarker, effectively increased the serum sensitivity to multiple *K. pneumoniae* clinical isolates and restored the bactericidal activity of complement. The *in vivo* synergistic effect of proline was validated in a murine infection model. Furthermore, we demonstrated that proline activates the pyruvate/TCA cycle, increases proton motive force, and enhances complement proteins binding to bacterial surface, forming membrane attack complex to kill serum-resistant *K. pneumoniae*. Our findings provide new insights for the development of metabolism-based approach to manage *K. pneumoniae* serum resistance and offer potential targets and strategies for host immunity-based anti-infection therapies.

## Introduction

*Klebsiella pneumoniae* is a Gram-negative bacterial pathogen that is closely associated with nosocomial infections [1]. This pathogen causes the highest mortality rate among common blood infection pathogens, and of particular concern is neonatal sepsis that has become the leading cause of infectious deaths in children [2,3]. *K. pneumoniae* is able to rapidly proliferate in the blood causing bloodstream infection (BSI), a critical systemic condition characterized by a broad spectrum of clinical manifestations, including but not limited to high fever, chills, tachycardia, and tachypnea [4]. In its most severe form, BSI can precipitate shock and multi-organ dysfunction syndrome. Thus, *K. pneumoniae* is a highly pathogenic bacteria that endangers human lives, which requires urgent attention to develop effective control strategy. Although antibiotics are the most effective way to treat *K. pneumoniae* infection, the emergence of multidrug-resistant *K. pneumoniae* isolates that are resistant to most first-line antibiotics highlights the significant challenges in treating this pathogen [5,6]. Alternative methods should be developed simultaneously.

Anti-virulence is becoming another attractive strategy to combat bacterial infection that disrupts bacterial virulence factors instead of essential genes, making bacteria less prone to evolve resistance [7]. The key virulent feature of *K. pneumoniae* is that it can evade complement-mediated killing, known as serum resistance, a prerequisite for successfully establishing BSI. Being consistent, isolates from clinical samples exhibited serum resistance [8]. Research on mechanisms of serum resistance includes inhibition of complement activation cascade and/or altering bacteria surface structure. The mechanisms of action on inhibiting complement activation can be summarized in several aspects: recruiting or mimicking complement regulatory proteins to render the complement system ineffective, modulating or inhibiting complement-binding proteins through interactions, and secreting enzymes to degrade complement components [9–11]. Some pathogens also evade complement-mediated killing by altering their surface structural features [12,13], for example, it has been demonstrated that the bacterial capsule and peptidoglycan can inhibit phagocytosis mediated by the complement system. Regardless of inhibition of complement activation or altering surface structure, the consequence is that formation of the membrane attack complex (MAC), the effector in killing bacteria, is failed. Although these mechanisms shed light on how bacteria evade complement system, few control measures are developed based on these mechanisms.

Recently, a large body of evidence suggest that bacterial metabolic state determines its sensitivity to serum-mediated killing, while altering bacterial metabolism could revert serum resistance [14]. In *Escherichia coli*, the metabolic pathway serine, glycine, and threonine metabolism were downregulated, leading to the upregulation of ATP, which in turn decreases the expression of complement binding proteins, abrogating complement deposition in bacterial surface. However, exogenous glycine could activate the metabolic pathway, repress ATP production, and enable CRP/cAMP in controlling the complement-binding protein expression [15]. In *Vibrio alginolyticus*, mannitol and glycine revert serum resistance through glycolysis and glutathione metabolism, respectively, that enhance complement deposition in bacterial surface [16]. Similarly, leucine can promote the serum killing of *Streptococcus agalactiae* [17]. All these studies suggest the importance of remodeling bacterial metabolism could be a potential approach to combat *K. pneumoniae* infection. In this study, we employed metabolomics approaches to systematically investigate the metabolic response of *K. pneumoniae* to serum exposure, uncover the key metabolic pathways and identify potential metabolites in potentiating serum to kill serum-resistant *K. pneumoniae*, providing a framework for therapeutic development.

## Results

### *Klebsiella pneumoniae* TS01 is resistant to serum-mediated killing

**Figure 1.**
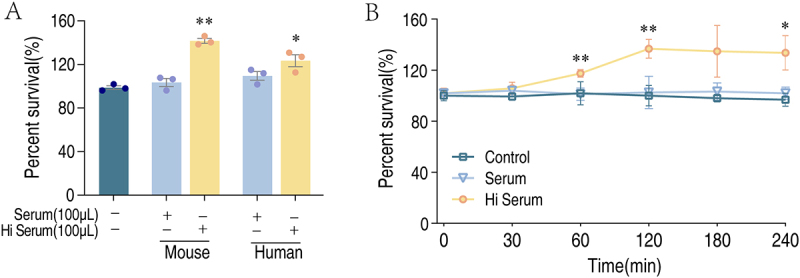
Percent survival of *K. pneumoniae* in serum and Hi serum. (A) Percent survival of *K. pneumoniae* TS01 in mouse or human serum and Hi serum. (B) Percent survival of *K. pneumoniae* TS01 in 100 μL mouse serum or in Hi mouse serum for the indicated time points. Results are displayed as mean ± standard errors of the means (SEM) (*N* ≥ 3 technical replicates per sample), and statistically significant differences are identified (*, *p* < 0.05; **, *p* < 0.01) as determined by Student’s *t* test.

*K. pneumoniae* TS01 is a clinic isolate from the serum of hospitalized patient. The sensitivity of TS01 to serum was investigated by incubating this strain with serum or heat-inactivated serum (Hi serum) from mouse or human, where the group treated without serum is the control group. Of notice, the bacteria strains were cultured in the absence of antibiotics. The viability of TS01 was slightly increased in either the mouse or human serum as compared to the control. However, the growth was greatly increased in Hi serum (Figure 1(A)). To further investigate the sensitivity of TS01 to serum, the bacteria was incubated with serum or heat-inactivated serum for different time points. Interestingly, the viability of bacteria in heat-inactivated serum was increased at the first 120 min and maintained in the following time, whereas the viability was unchanged at all time points if incubated in serum (Figure 1(B)). These data together suggest that *K. pneumoniae* TS01 is resistant to serum killing.

### Metabolic shifts of *Klebsiella pneumoniae* exposed to active serum and heat-inactivated serum

To investigate the underlying mechanism of how TS01 cope with serum-induced stress, bacteria were treated with serum or Hi serum for 2 hours, and then collected for mass spectrometry metabolomics. Each treatment included three biological replicates, and each biological replicate had two technical replicates, yielding 18 data points in total. Correlation coefficients are between 0.991 and 0.999, indicating the data was reproducible among replicates (Supplementary Figure S1A). To identify metabolites, the internal standard and solvents peaks were excluded. At last, 67 metabolites were identified. Clustering analysis showed the replicates of control, serum and Hi serum were separately clustered, indicating bacteria mounts differential metabolism to the treatments (Supplementary Figure S1B). Furthermore, the 67 metabolites can be classified into different categories, where 20.89%, 32.84%, 29.85%, 10.45%, and 5.97% of the metabolites are carbohydrates, amino acids, lipids, nucleotides, and others, respectively (Supplementary Figure S1C).

**Figure 2.**
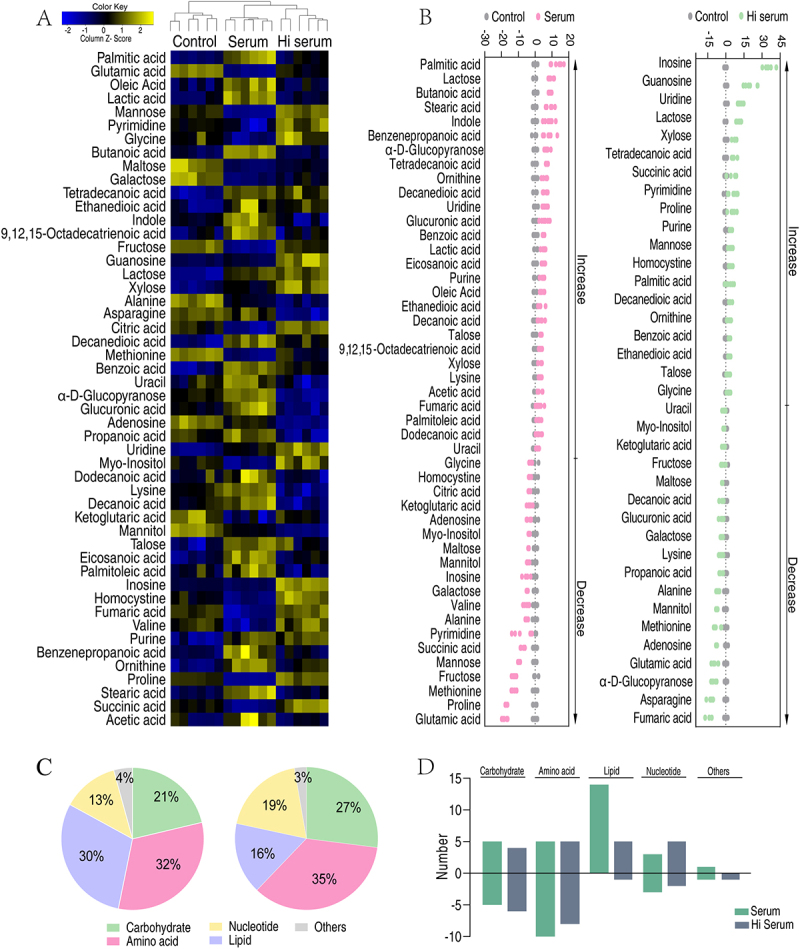
Metabolomic profiling of the serum- and Hi serum-treated groups. (A) Heat map showing differential metabolites. Yellow and blue colors indicate increase and decrease of metabolites relative to the median metabolite level, respectively (see color scale). (B) Z-score plot of differential metabolites based on the control. The data from the serum (left) and the Hi-serum (right) groups were scaled to the mean and standard deviation of the control group. Each point represents one metabolite in one technical repeat and is colored by sample type (gray, control; pink, serum; red, heat-inactivated serum). (C) Category of the differential abundance of metabolites in the serum group (left) and Hi serum group (right). (D) The number of metabolites increased and decreased in each of the functional categories.

Next, we employed Kruskal–Wallis test to identify differential metabolites. Compared to the control group, 47 and 37 differential metabolites were identified (*p*<0.05) in the serum- and Hi serum-treated groups, respectively (Figure 2(A)). Z-values spanned from −18.29 to 12.97 in the serum-treated group, and from −13.92 to 35.47 in Hi serum-treated group. Accordingly, the number of increased and decreased abundance of metabolites are 28 metabolites and 19 metabolites in serum-treated group, respectively; 19 metabolites and 18 metabolites in Hi-treated group, respectively (Figure 2(B)).

Functional categories of the differential metabolites were analyzed. Interestingly, amino acids comprised the largest portion of differential metabolites in both the serum-treated and Hi serum-treated groups, being 32% and 35%, respectively, suggesting amino acid metabolism may actively engaged in bacterial response to serum (Figure 2(C)). In addition, the serum-treated and heat-inactivated serum-treated groups exhibited similar numbers of altered metabolites across all categories except for lipids, where the number of increased metabolites was higher in serum-treated group as compared to Hi serum-treated group (Figure 2(D)). These results indicate that bacteria alter their metabolism in response to serum-induced stress.

### Pathway analysis of differential metabolites after treatment with active and heat-inactivated serum

**Figure 3.**
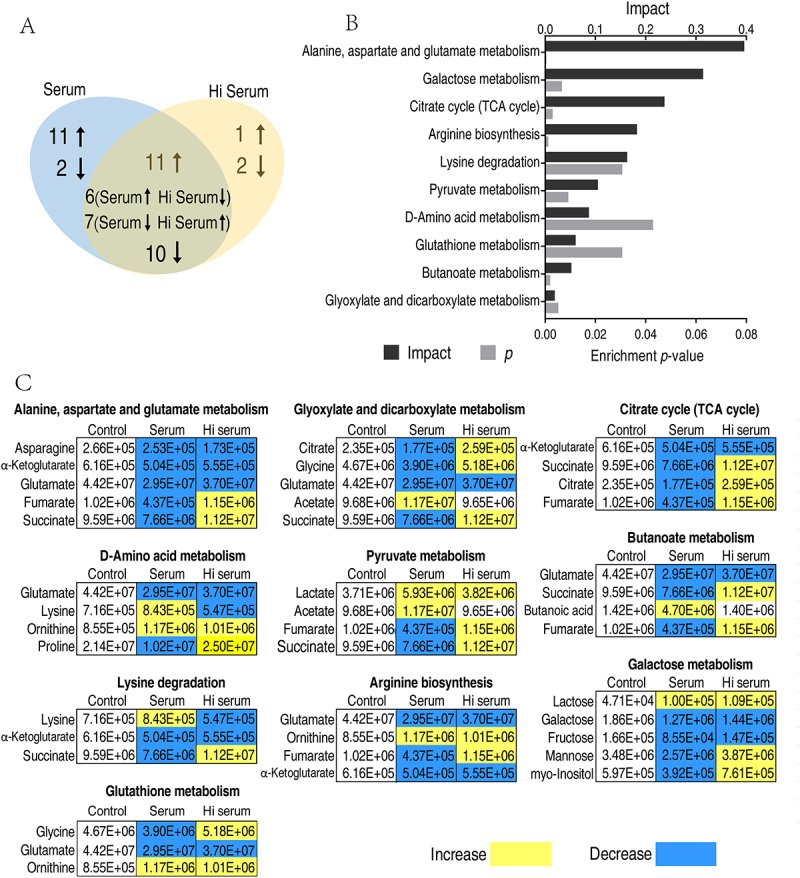
Pathway enrichment analysis of differential metabolites. (A) Venn diagram showing the overlapping and unique differential metabolites between the serum group and the Hi serum group. Decreased and increased metabolites are indicated with down and up arrows, respectively. (B) Pathway enrichment analysis of differential metabolites. Significantly enriched pathways (*p*-value < 0.05) were selected for plotting and the impact scores. (C) Integrative analysis of metabolites in enriched pathways. Yellow and blue colors indicate increased and decreased metabolites, respectively.

The differential metabolites were further analyzed with pathway enrichment analysis. Among these differential metabolites, 34 metabolites were overlapped, 10 metabolites were decreased, 11 were increased, and 13 showed differential abundance between the two groups. The others were specific to serum- or Hi-serum-treated groups, whereas 11 increased metabolites and 2 decreased metabolites in the serum-treated group, and 1 increased metabolite and 2 decreased metabolites in Hi serum-treated group (Figure 3(A)). Ten pathways were enriched (*p* < 0.05), including alanine, aspartate and glutamate metabolism, galactose metabolism, citrate cycle (which was part of the pyruvate cycle [18], we referred it as pyruvate/TCA cycle afterward), arginine biosynthesis, lysine degradation, pyruvate metabolism, D-amino acid metabolism, glutathione metabolism, butanoate metabolism and glyoxylate and dicarboxylate metabolism (Figure 3(B)). It is noteworthy that the abundance of most differential metabolites was downregulated in the serum group, especially in the Pyruvate/TCA cycle and alanine, aspartate, and glutamate metabolism, among which the abundance of metabolites were decreased (Figure 3(C)). This supports our previous investigation that the central carbon metabolism is significantly downregulated when bacteria are exposed to serum [15,19]. When the complement is inactivated, the abundance of most differential metabolites showed opposite change as that in the serum group. This observation suggests that the differential metabolic response of bacteria to serum and Hi serum is dependent on complement.

### Identification of crucial biomarkers that distinguish serum- and Hi serum-treated groups

**Figure 4.**
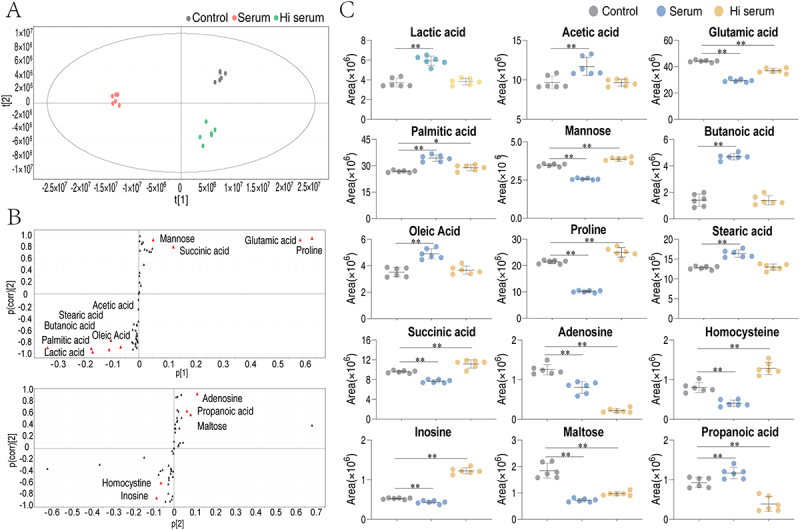
Identification of crucial biomarkers. (A) PCA analysis of the control group, the serum- and Hi-treated groups. Each dot represents a technical replicate of the samples in the plot. (B) S-plot generated from OPLS-DA. Triangles represent individual metabolites, where potential biomarkers are highlighted in red if their absolute values of covariance *p* and correlation p(corr) are greater than or equal to 0.05 and 0.5, respectively. (C) Scatter diagram of 15 biomarkers. *, *p* < 0.05; **; *p* < 0.01.

To identify biomarkers that represent the could separate the serum- and Hi serum-treated groups, OPLS-DA was employed to discern metabolomic patterns of the samples. By such analysis, the control, serum- and Hi serum-treated groups were clearly separated and clustered with their own (Figure 4(A)), indicating they have their own metabolic signatures. Component t [1] differentiated the serum-treated group from the control and Hi serum-treated group, while component t [2] specifically differentiated the Hi serum-treated group from the other two groups. The discriminant variables were identified using S-plots, with cutoff values set to an absolute covariance p-value of ≥0.05 and a correlation p-value (corr) of ≥0.5 (Figure 4(B)). This analysis resulted in the identification of 10 biomarkers (proline, glutamic acid, mannose, succinic acid, acetic acid, stearic acid, butanoic acid, oleic acid, lactic acid, and palmitic acid) based on the correlation between p [1] and p (corr) [1] (Figure 4(C)). Additionally, five biomarkers (adenosine, propanoic acid, maltose, homocysteine, and inosine) were identified based on the correlation between p [2] and p(corr) [2]. The abundance of five metabolites (mannose, proline, succinic acid, homocysteine, and inosine) were decreased in serum-treated group but increased in Hi serum-treated group. Noteworthy, proline had the highest covariance *p* value and the greatest difference in abundance among the control, serum- and Hi serum-treated groups. The significant changes in proline abundance under different serum conditions highlight its potential as a key mediator in bacterial responses to serum treatment. Moreover, proline belongs to the D-amino acid metabolism that was enriched in pathway enrichment analysis (Figure 3(C)). Taken together, we selected proline for the following functional studies.

### Exogenous proline enhances the sensitivity of *K. pneumoniae* to serum

**Figure 5.**
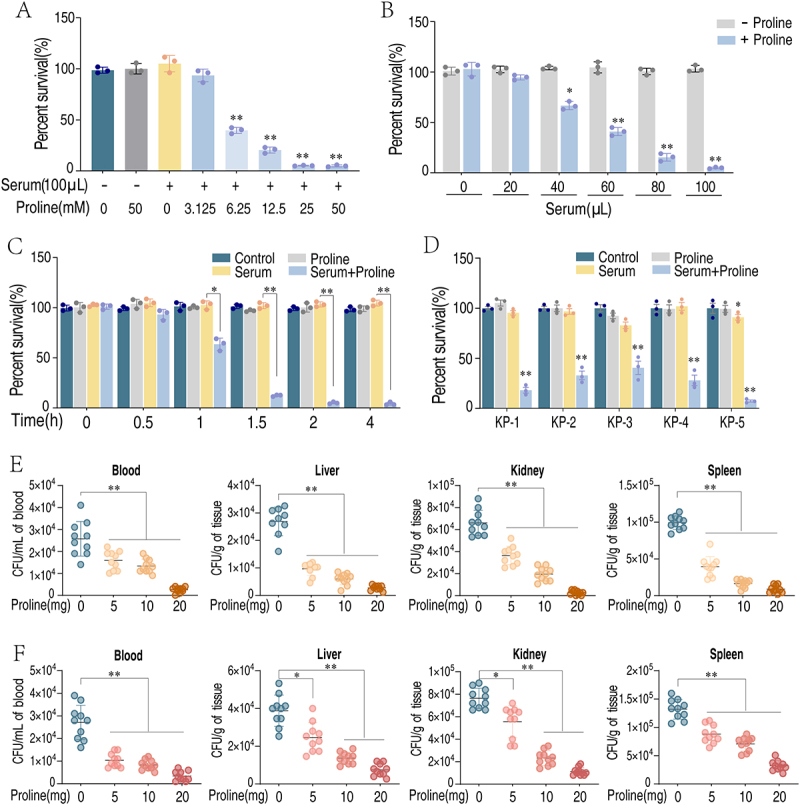
Exogenous proline enhances the sensitivity of *K. pneumoniae* to serum-induced killing. (A) Percent survival of *K. pneumoniae* TS01 in the indicated concentrations of proline plus 100 μL mouse serum. (B) Percent survival of *K. pneumoniae* TS01 in the indicated concentrations of mouse serum (0–100 μL) plus 25 mM proline. (C) Percent survival of *K. pneumoniae* TS01 in 25 mM proline plus 100 μL mouse serum over the indicated incubation times. (D) Percent survival of clinically isolated *K. pneumoniae* strains in the presence of 25 mM proline, 100 μL serum, and 25 mM proline plus 100 μL serum. (E) BALB/c mice were injected i.p. with the indicated bacteria and treated with proline as described. Bacterial load was measured in blood, liver, kidney, and spleen (*n* = 10). (F) BALB/c mice were injected i.v. with the indicated bacteria and treated with proline as described. Bacterial load was measured in blood, liver, kidney, and spleen (*n* = 10). Results are displayed as mean ± standard errors of the means (SEM) (*N* ≥ 3 technical replicates per sample), and statistically significant differences are identified (*, *p* < 0.05; **; *p* < 0.01) as determined by Student’s *t* test.

The metabolic state of bacteria is decisive in their sensitivity to serum killing [15,20]. Serum-sensitive strains exhibit significant differences in metabolic characteristics compared to serum-resistant strains. By employing metabolite reprogramming strategies, it is possible to transform the metabolic state of serum-resistant bacteria into a sensitive state, thereby restoring their sensitivity to serum complement [15,16,21]. To verify this hypothesis, we performed serum killing experiments in *K. pneumoniae*. When bacteria was treated with serum at 100 μL, proline was added sequentially at concentrations of 0, 3.125, 6.25, 12.5, 25, and 50 mM. Proline alone had no effect on *K. pneumoniae* survival upon serum treatment, but when combined with serum, the bacterial survival rate decreased from 100% to 4.71% as the concentration of proline increased (Figure 5(A)). Then, bacteria were treated with proline (25 mM) plus various concentrations of serum at 0, 20, 40, 60, 80, and 100 μL. The survival rate decreased from 100% to 5.11% (Figure 5(B)).

Finally, a time-dependent experiment was performed using 25 mM proline combined with 100 μL serum. The data showed that the presence of proline over time enhanced the bactericidal activity of serum, leading to a more pronounced reduction in bacterial survival, confirming the time-dependent promoting effect of proline on serum killing (Figure 5(C)). The above results suggest that exogenous proline reversed *K. pneumoniae* resistance and restore the bactericidal activity of serum. To ensure that this effect is not strain-specific, five clinical isolates of *K. pneumoniae* were tested. These isolates could grow in Hi serum but were slightly affected by serum (Figure 5(D)). The presence of proline significantly enhanced serum killing that reduced bacterial survival from 40.51% to 7.44% (Figure 5(D)). This consistent effect across multiple strains suggests that the ability of proline to enhance serum killing is not limited to a specific isolate. To exclude the possible bactericidal effect of proline, bacteria were treated with Hi serum or Hi serum plus proline, where proline had no effect on bacterial viability (Supplementary Figure S2).

To further validate the in *vivo* effects of proline in promoting serum killing of bacteria, we employed two distinct infection models. BALB/c mice were intraperitoneally injected with proline (5, 10, or 20 mg per mouse) twice daily for 2 days, while mice injected with saline only was control. After last injection, mice were infected with 1 × 10^6^ CFU of *K. pneumoniae* administered through intraperitoneal injection or intravenous injection through tail vein. Bacterial counts were monitored in blood, liver, spleen, and kidney samples. Bacterial loads in these four organs decreased as the dose of proline increased. Specifically, the 20 mg/kg dose of proline had the most significant reduction in bacterial loads with both application routes (Figure 5(E,F)). These data demonstrate that proline reverses *K. pneumoniae* serum resistance both *in vitro* and *in vivo*.

### Exogenous proline promotes complement components depositing to bacterial surface

**Figure 6.**
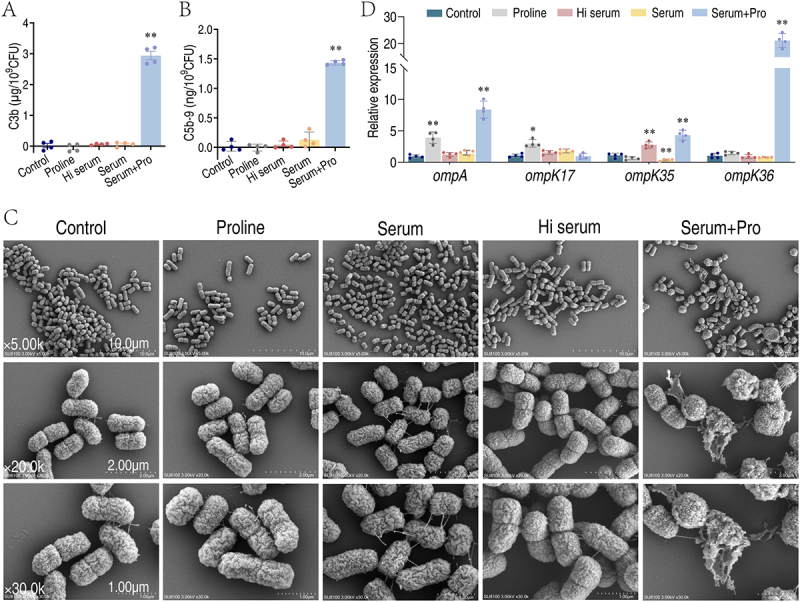
Exogenous proline increases serum complement binding to the bacterial surface. (A & B) Quantification of C3b (A) and C5b-9 (B) incubated with 25 mM proline, 100 μL heat-inactivated serum, 100 μL serum, and 25 mM proline plus 100 μL serum. (C) Scanning electron microscopy (SEM) of *K. pneumoniae* in the presence of 25 mM proline, 100 μL heat-inactivated serum, 100 μL serum, and 25 mM proline plus 100 μL serum. (D) Real-time quantitative reverse transcription-PCR (qRT-PCR) for quantifying the gene expression of outer membrane proteins. Results are displayed as mean ± standard errors of the means (SEM) (*N* ≥ 3 technical replicates per sample), and statistically significant differences are identified (*, *p* < 0.05; **, *p* < 0.01) as determined by Student’s *t* test.

Complement binding to bacterial surface is crucial to form membrane attack complex (MAC), which disrupts the bacterial membrane, leading to the leakage of intracellular contents and ultimately causing bacterial death. Thus, the binding of complement components to bacteria is key to serum killing. Interestingly, *K. pneumoniae* treated with serum alone had few complement components being detected on the bacterial surface, while proline increased the binding of C3b and C5b-9 in bacterial surface (Figure 6(A,B)). To further confirm the results, scanning electron microscopy (SEM) was used to examine the impact of exogenous proline supplementation on the surface morphology of *K. pneumoniae* cells (Figure 6(C)). *K. pneumoniae* displayed as short, rod-shaped with a rough and wrinkled surface [22]. Compared to the non-treated group, proline, serum, or Hi serum did not alter the surface structure or morphology of the bacteria; however, when both proline and serum were present, most bacteria lost their original cell shape, appearing more spherical, while some showed clear rupture on cell membrane (Figure 6(C)).

Moreover, OmpA, OmpK17, OmpK35, and OmpK36 are known complement binding proteins in *K. pneumoniae* [23–27]. To investigate the impact of proline on these outer membrane proteins, we quantified the expression of genes with qRT-PCR. As compared to control, exposure of bacteria to Hi serum slightly increased *ompK35* expression, while proline alone slightly increased *ompA* and *ompK17* expression (Figure 6(D)). Interestingly, the combination of both serum and proline significantly increased *ompA*, *ompK35* and *ompK36* expression (Figure 6(D)). This finding suggests that proline enhances complement-mediated cell lysis.

### Exogenous proline promotes serum complement-mediated killing of *K. pneumoniae* via the pyruvate cycle

**Figure 7.**
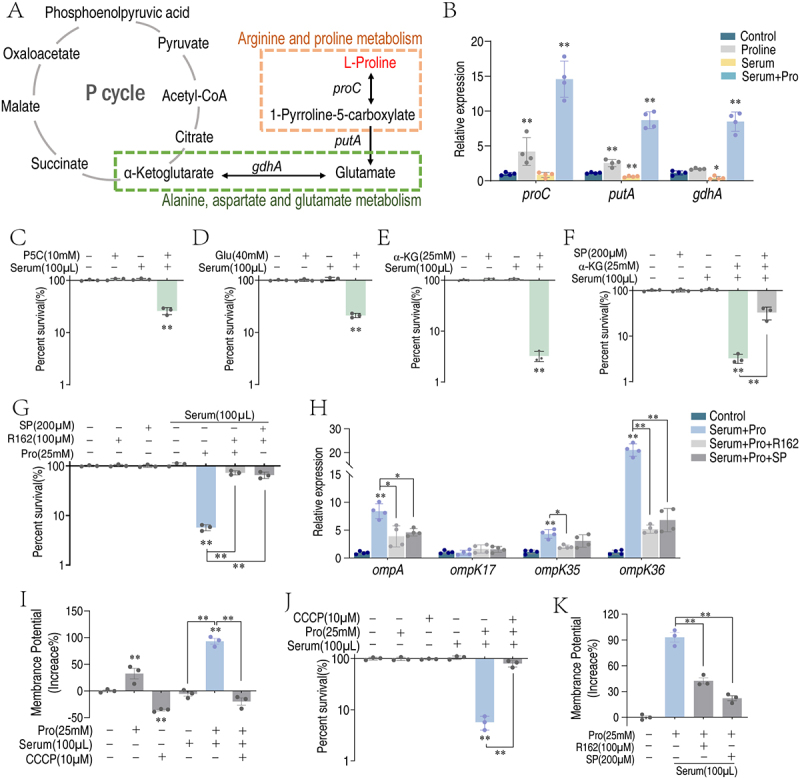
Proline promotes the pyruvate cycle in *K. pneumoniae*. (A) Proline metabolism. (B) qRT-PCR for quantifying the expression of genes downstream of proline metabolism. (C-E) Percent survival of *K. pneumoniae* TS01 in the presence of the indicated concentration of pyrrolidine-5-carboxylate (P5C) (C), glutamate (Glu) (D), and α-ketoglutarate (α-KG) (E) plus 100 μL serum. (F) Percent survival of *K. pneumoniae* TS01 in the presence of 25 mM α-KG plus 100 μL serum, and the effect of 200 μM succinyl phosphonate (SP). (G) Percent survival of *K. pneumoniae* TS01 in the presence of 25 mM proline (Pro) plus 100 μL serum, and the effect of 200 μM SP or 100 μM R162. (H) qRT-PCR for quantifying the gene expression of outer membrane proteins in the presence of 25 mM proline plus 100 μL serum, or in the presence of 200 μM SP or 100 μM R162. (I) Membrane potential of *K. pneumoniae* TS01 treated with 100 ml serum in the presence or absence of 25 mM proline or/and 10 μΜ CCCP. (J) Percent survival of *K. pneumoniae* TS01 in the presence of 25 mM Pro plus 100 μL serum, and the effect of 10 μΜ CCCP. (K) Membrane potential of *K. pneumoniae* TS01 in the presence of 25 mM Pro plus 100 μL serum, and the effect of 200 μM SP or 100 μM R162.Results are displayed as mean ± standard errors of the means (SEM) (*N* ≥ 3 technical replicates per sample), and statistically significant differences are identified (*, *p* < 0.05; **, *p* < 0.01) as determined by Student’s *t* test.

To investigate the possible route that proline affects the expression of complement binding proteins, we trace the metabolism of proline. Proline can be metabolized into pyrrolidine-5-carboxylate (P5C), glutamate, and α-ketoglutarate, ultimately entering the pyruvate cycle. Specifically, the conversion of proline to P5C is catalyzed by proline dehydrogenase (encoded by the *proC* gene). The subsequent steps involve the action of two additional enzymes: P5C dehydrogenase (*putA*) converts P5C into glutamate, and glutamate dehydrogenase (*gdhA*) facilitates the transformation of glutamate into α-ketoglutarate (Figure 7(A)). Proline treatment increased the expression of *proC* and *putA*, while serum treatment alone decreased the expression of *gdhA*. When bacteria were treated with proline and serum, the expression of *proC*, *putA*, and *gdhA* was significantly increased (Figure 7(B)), suggesting proline flux into the pyruvate cycle. Additionally, supplementation of downstream metabolites of proline, P5C, glutamate, and α-ketoglutarate also enhanced serum killing toward *K. pneumoniae*, where α-ketoglutarate had the best synergistic efficacy (Figure 7(C-E)). Treatment of bacteria with R162, an inhibitor of glutamate dehydrogenase encoded by *gdhA*, and SP, an inhibitor of α-ketoglutarate dehydrogenase that converts α-ketoglutarate to succinate, abolished proline-enabled serum killing (Figure 7(G)). Being consistent, the presence of SP also reduced the promoting effect of α-ketoglutarate (Figure 7(F)). Moreover, the treatment of bacteria with either R162 or SP decreased the expression of complement binding proteins (Figure 7(H)).

Pyruvate cycle is known to provide energy and generate proton motive force (PMF) in bacteria [18,28,29]. To investigate whether PMF was involved in proline-enabled serum killing, bacteria were treated with proline, serum, or both, in the presence of carbonyl cyanide m-chlorophenyl hydrazine (CCCP) [30]. Proline alone slightly increased PMF, but serum alone did not. However, the combination of proline and serum significantly boosted PMF, which was abrogated by CCCP (Figure 7(I)). Consistent with the PMF results, CCCP inhibited proline-enabled serum killing (Figure 7(J)). Similarly, the inhibitors R162 and SP reduced the PMF triggered by proline, corroborating that proline fluxes into the pyruvate cycle to support serum killing (Figure 7(K)). These results suggest that proline enhances serum killing through upregulating the expression of complement binding proteins and increases the generation of proton motive force.

## Discussion

Deaths caused by bacterial infections have become the second leading cause of death globally, posing a significant challenge to healthcare and public health. Among these deaths, more than one-third are attributed to bloodstream infections [4,6]. Serum resistance, an important pathogenic trait, allows pathogens to evade immune clearance by the host’s complement system, thereby enabling their survival in the bloodstream [31]. Most clinically isolated pathogenic bacteria display resistance to the serum complement and exploit nutrients in the blood for rapid proliferation [32,33]. Addressing serum resistance is essential for mitigating the disease burden associated with bacterial infections. Recent studies increasingly indicate that targeting metabolic regulation might provide novel approaches and strategies to combat bacterial serum resistance [8,15,20,34]. Metabolic reprogramming can act as an effective method to remodel bacterial metabolic state and reinstate its susceptibility to serum complement [21,35,36].

In this study, we found that *K. pneumoniae* alters its metabolic state in response to complement with a significant downregulation of the pyruvate/TCA cycle. This finding aligns with previous observations that the downregulated central carbon metabolism is a key characteristic of serum-resistant *E. coli* [15,19]. Additionally, we observed a significant downregulation of the alanine, aspartate, and glutamate metabolism pathways in serum-treated group. Interestingly, exogenous supplementation with proline activates the pyruvate cycle through the alanine, aspartate, and glutamate metabolic pathways. Activation of the pyruvate/TCA cycle enhances PMF, which in turn promotes the expression of outer membrane proteins OmpA, OmpK35, and OmpK36 [23,24,37]. The PMF provides the driving force for the effective binding of complement proteins to outer membrane proteins, thereby increasing the bactericidal efficiency of the complement.

Proline plays diverse roles in bacteria [38]. It not only serves as a precursor for protein synthesis but is also converted to glutamate via the proline dehydrogenase and proline oxidase pathways, subsequently entering the Pyruvate/TCA cycle to participate in energy metabolism. Previous studies have demonstrated that many bacteria accumulate proline under conditions of high osmotic pressure, oxidative stress, antibiotic exposure, and other unfavorable environments to enhance their survival. For instance, *Staphylococcus aureus* accumulates proline under high-salt conditions, thereby bolstering its resistance to environmental stress [39]. Similarly, *Pseudomonas aeruginosa* accumulates proline during biofilm formation, which not only helps maintain intracellular stability but also increases virulence and antibiotic resistance by modulating the biofilm structure and function [40]. Although the role of proline in bacterial biology has been extensively studied, its involvement in bacterial serum resistance has not been reported. Interestingly, our study found that exposure to serum environments results in a reduction of both proline abundance and proline metabolism in bacteria. Notably, proline was undetected in serum-resistant *E. coli* strains [15]. This absence could potentially be attributed to technical limitations in metabolite identification during our previous investigation. However, we cannot exclude the possibility that proline might possess broad-spectrum potentiating effects on serum killing. Our findings suggest that proline enhances serum bactericidal activity primarily through modulation of alanine, aspartate and glutamate metabolism and the pyruvate cycle, two pathways present across diverse bacterial species. Meanwhile, we should be aware that this potentiating effect may exhibit interspecies variability, particularly given the incomplete understanding of proline’s role in regulating complement-binding protein expression. Further studies can be performed to test those possibilities. Together, these findings suggest that bacterial metabolic states vary under different environmental stresses, from which we can identify the key metabolites being influenced.

Proline has a wide range of clinical applications, serving as one of the components in compound amino acid large-volume infusions [41]. Proline is used in conditions including malnutrition, protein deficiency, severe gastrointestinal diseases, burns, and post-surgical protein supplementation, with no significant toxic or side effects [42,43]. Proline stabilizes IgG production by reducing dimers and aggregates formation, enabling long-term storage at room temperature to ensure product stability and safety. The IgG dimers and aggregates may trigger systemic reactions [44,45]. Clinical data show that after treatment with Privigen or Hizentra (proline-stabilized immunoglobulin products), proline does not accumulate and is not associated with adverse reactions [46]. The maximum tolerated dose of proline in humans with normal proline metabolic function is currently unknown. However, while the maximum recommended dose for Privigen in humans contains 575 mg proline/kg [44], the 250 mg proline/kg dose we used in mice (less than 50% of the human dose) was sufficient to clear *K. pneumoniae* infection. Therefore, proline demonstrated safety and feasibility as a potential therapeutic candidate exhibiting favorable safety and feasibility profiles for counteracting pathogen serum resistance. Its translational potential could be explored as a parenteral administration regimen or as an adjunctive therapeutic component in combination therapies to treat *K. pneumonia* bloodstream infection.

Using metabolites that bacteria can utilize for the prevention and treatment of bacterial infections is a safe and effective strategy [47–51]. Exogenous supplementation of glucose or alanine can restore the sensitivity of resistant bacteria to kanamycin [30]. Exogenous glutamine activates the glutamine-adenosine-CpxA/CpxR-OmpF regulatory pathway, promoting bacterial uptake of common antibiotics and efficiently clearing clinical isolates of multidrug-resistant bacteria, including *K. pneumonia*, both *in vitro* and *in vivo* [52]. Exogenous glycine can inhibit ATP synthase, leading to intracellular ATP depletion, which reduces the production of the transcription factor cAMP/CRP and promotes the expression of complement-binding proteins [15]. Exogenous metabolites such as succinate and inosine competitively modulate both cellular and humoral immune processes, affecting the host’s immune response to bacterial infections [53]. Exogenous L-glutamine enhances bactericidal activity of aminoglycoside antibiotics (e.g. gentamicin) against Gram-positive resistant bacteria [54]. Existing studies also demonstrated the efficacy of metabolic strategies in revealing, identifying, and analyzing bacterial pathogenic features for disease treatment and prevention.

**Figure 8.**
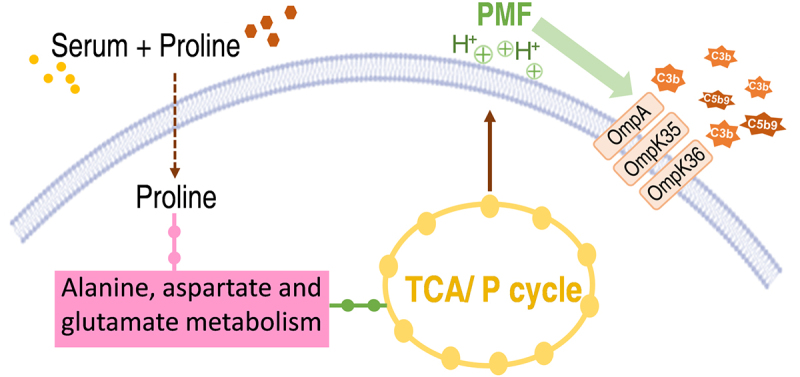
Proposed model. In the presence of serum, exogenous proline, through alanine, aspartate, and glutamate, activates the pyruvate cycle. This action not only increases the proton motive force (PMF) but also enhances the binding of complement proteins to outer membrane proteins OmpA, OmpK35, and OmpK36, facilitating the formation of complement-mediated membrane attack complexes.

In summary, this study demonstrates that proline is a potent metabolite in reversing *K. pneumoniae* serum resistance both in *vitro* and in *vivo* by fluxing into the pyruvate/TCA cycle. Our data show a correlated upregulation of OmpA/OmpK35/OmpK36 expression with increased C3b deposition and MAC formation upon proline treatment (Figure 8). Although the exact mechanistic link requires further investigation, these observations suggest a potential alternative approach to combat *K. pneumoniae* infection in an antibiotic-independent manner.

## Materials and methods

### Ethics statement

#### Human research ethics

This study utilized de-identified *Klebsiella pneumoniae* clinical isolates obtained from residual diagnostic specimens of bloodstream infection patients at the Affiliated Cancer Hospital of Guangzhou Medical University (Guangzhou, China). This study was conducted in strict compliance with the Declaration of Helsinki (https://www.wma.net/policies-post/wma-declaration-of-helsinki/) and was approved by the Medical Ethics Committee of the Affiliated Cancer Hospital, Guangzhou Medical University (approval no. KY-2024023-01). The ethics committee specifically reviewed and approved both the electronic consent procedure and the study protocol. Prior to sample collection, electronic informed consent was obtained through the hospital’s certified online registration system, where patients voluntarily opted to authorize the use of de-identified residual clinical specimens for research purposes. Isolates were de-identified and preserved at −80°C in the clinical microbiology laboratory’s secure storage system prior to research use, following institutional biosafety protocols.

#### Animal research ethics

Animal experiments were approved by the Institutional Animal Care and Use Committee (IACUC) of Sun Yat-sen University (approval no. SYSU-IACUC-2023-B0538). All procedures, including euthanasia, were conducted in compliance with the AVMA Guidelines for the Euthanasia of Animals (2020) and the NIH Guide for the Care and Use of Laboratory Animals. All animal procedures followed the ARRIVE guidelines 2.0 (the complete checklist is available at Figshare: https://doi.org/10.6084/m9.figshare.29083565.v3).

### Bacterial strains and culture conditions

The serum-resistant *Klebsiella pneumoniae* strains (clinical origin detailed in Ethics Statement) were isolated from bloodstream infection patients during standard diagnostic procedures at the Affiliated Cancer Hospital of Guangzhou Medical University. Bacterial stocks were preserved in LB medium containing 25% glycerol at −80°C, with viability assessments conducted every 6 months. For the experiments, glycerol stocks of the strains were streaked onto LB agar plates and incubated overnight at 37°C. Subsequently, single colonies were inoculated into fresh Luria−Bertani (LB) (tryptone 1%, yeast extract 0.5%, and NaCl 1%) broth, and cultured at 37°C, 200 rpm.

### Serum killing

Serum killing was carried out as described previously [15,20]. Bacteria in the logarithmic growth phase were collected, washed with sterile saline, and resuspended to adjust the OD600 to 1.0. The bacterial suspension was aliquoted into sterile centrifuge tubes, and the bacteria were pelleted by centrifugation. The following treatments were applied to the bacteria in four groups: saline only, serum only, metabolites only, and metabolites plus serum. Each treatment was performed in triplicate. Following incubation, serial dilutions were performed, and bacterial samples were spread onto solid agar plates (using at least three dilution steps). After overnight incubation, the colony-forming units (CFU/mL) were enumerated. The bacterial survival rate was determined by comparing the number of viable bacteria in the serum-only or metabolites plus serum treatment groups to that of the saline control group.

### Metabolomic profiling

GC-MS sample preparation and analysis were conducted as previously described [20,35]. Bacterial cells were incubated with serum or heat-inactivated serum for 2 h at 37°C and then re-suspended in saline to achieve an OD600 of 1.0. The bacterial suspension (10 mL) was centrifuged to collect the bacterial pellet. Intracellular metabolites were extracted using 1000 μL of ice-cold methanol, and 10 μL of a 0.1 mg/mL ribitol solution was added as an internal standard. The cells were disrupted by sonication at 30% intensity for 10 min, followed by centrifugation at 12,000 g for 10 min at 4°C. Next, 1000 μL of the supernatant was transferred to a fresh 1.5 mL tube, and the methanol was evaporated from the extract to prepare for GC-MS analysis. Then, 80 μL of a 20 mg/mL solution of methoxyamine hydrochloride in pyridine (Sigma-Aldrich) was added to the metabolite samples and incubated at 37°C for 3 h. Following this, 80 μL of N-methyl-N-trimethylsilyltrifluoro-acetamide (MSTFA) was added, and the mixture was shaken at 37°C for 30 min. The samples were then centrifuged to obtain the supernatant, which was analyzed using an Agilent 7890A gas chromatograph coupled and an Agilent 5975C Inert XL mass selective detector (Agilent Technologies). Chromatographic peak areas were extracted using Thermo Fisher’s XCalibur 4.0 software.

### GC-MS data analysis

The mass fragmentation spectra were analyzed using XCalibur software (Thermo Fisher, version 2.1) to identify compounds by comparing the data with the National Institute of Standards and Technology (NIST) library and using the NIST MS Search 2.0 program [29]. The data were normalized using an internal standard (ribitol) and the total metabolite content. The standardized data, encompassing metabolites, retention times, and peak areas, were utilized for subsequent metabolomics analysis. Significant differences in the standardized data were analyzed using IBM SPSS Statistics 19, and metabolites with *p*-values <0.05 were selected. Hierarchical clustering was conducted using R software. Principal component analysis (PCA) and orthogonal partial least squares discriminant analysis (OPLS-DA) were performed using SIMCA-P + 12.0 software (Umetrics, Umeå, Sweden). Pathway enrichment analysis for the differential metabolites was executed using MetaboAnalyst 5.0 [36,50].

### Bacterial clearance in a mouse model

*In vivo* validation of the effect of proline on reversing bacterial serum resistance was conducted according to the methods described previously [15]. Male BALB/c mice (7– 8 weeks, 20 ± 2 g) were obtained from the Laboratory Animal Center of Sun Yat-sen University (Guangzhou, China; animal production license no. SCXK 2021–0029). All animals meeting inclusion criteria (no physical/behavioral abnormalities after ≥5-d acclimation) were housed under specific pathogen-free conditions with 12:12 light-dark cycles and ad libitum access to food/water. The study employed 80 mice randomly allocated to two parallel infection route groups (*n* = 40 per route): intraperitoneal or tail vein intravenous injection. For each route, mice were stratified into four treatment groups (*n* = 10 per group): (1) saline-treated control, and proline-treated groups receiving (2) 5 mg per mouse, (3) 10 mg per mouse, or (4) 20 mg per mouse, with all treatments administered via intraperitoneal injection every 12 h for four consecutive doses. After the final treatment, mice were challenged with 1 × 10^6^ CFU *K. pneumoniae* via the respective routes. At 24 h post-infection, mice were anesthetized via intraperitoneal administration of sodium pentobarbital (50 mg/kg) and euthanized via cervical dislocation. Bacterial loads in blood and organs were quantified to evaluate clearance rates.

### Measurement of complement C3b/C5b-9 binding levels

The levels of complement fragment C3b and complement protein C5b-9 were measured using commercial assay kits [16,20]. Bacteria were treated according to experimental groups for 1.5 h, after which the bacterial cells were harvested and washed with sterile saline. The bacterial suspension was adjusted to an OD600 of 1.0. Then, 50 μL of the bacterial suspension was added to wells precoated with specific antibodies, and measurements were performed according to the instructions provided with the assay kits.

### Quantitative real-time PCR

Quantitative real-time PCR (qRT-PCR) was carried out as described previously [48,55]. Total RNA was extracted from bacteria (1 mL, OD600 = 1.0) using TRIzol reagent (Invitrogen, Life Technologies), followed by chloroform separation and isopropanol precipitation. Electrophoresis in 1% (wt/vol) agarose gels was performed to check the quality of extracted RNA. Reverse transcription was performed using the EvoM-MLV RT Kit with gDNA clean function (AG11705; Accurate Biotechnology) according to the manufacturer’s instructions. The procedure included: (1) elimination of residual genomic DNA, followed by (2) cDNA synthesis using 1 μg of total RNA as template. qRT-PCR was conducted using the SYBR Green Premix Pro Taq HS qPCR kit (AG11701; Accurate Biotechnology) on a 384-well optical plate, with a final reaction volume of 10 μL. The reactions were carried out on a LightCycler 480 system (Roche, Germany). The cycling conditions were as follows: 30 s at 95°C for polymerase activation, followed by 40 cycles of 5 s at 95°C for denaturation and 30 s at 60°C for annealing [48]. Data were analyzed using the 2^−ΔΔCT^ method, with 16S rRNA as the reference gene. The genes and primers used in this study are provided in Supplementary Table S1.

### Scanning electron microscopy (SEM)

Bacterial samples were collected and preserved overnight at 4°C in a 2.5% glutaraldehyde solution. The samples were then washed with 0.1 M phosphate-buffered saline (PBS, pH 7.0) and underwent post-fixation with 1% osmium tetroxide for 2 h. After an additional wash, the samples were dehydrated through a graded series of ethanol solutions. They were subsequently treated with a 1:1 mixture of ethanol and isoamyl acetate for 30 min, followed by immersion in pure isoamyl acetate for 1 h. The samples were then dried using critical point drying, sputter-coated with gold, and examined under a scanning electron microscope.

### Measurement of membrane potential

Membrane potential was measured as reported [28,56]. The bacterial cells were diluted to a concentration of 1 × 10^6^ CFU/mL in saline and then incubated with 10 μM DiOC2(3) for 30 min at 37°C. Subsequently, flow cytometric analysis was carried out using a FACSCalibur flow cytometer, with the instrument set to the following parameters: FITC channel voltage at 250 V, mCherry channel voltage at 650 V, forward scatter (FSC) threshold at 1,000, and a total of 10,000 events were recorded. The red/green (mCherry/FITC) fluorescence ratio for each cell was measured and normalized. The membrane potential was calculated using the formula: membrane potential = log(10^3/2^ × red fluorescence/green fluorescence).

### Statistical analysis

Data analysis and visualization were performed using GraphPad Prism 8.0. After assessing the normality of the data, pairwise comparisons were conducted using a two-tailed paired Student’s *t* test, unless otherwise indicated. The results are expressed as means ± standard error of the mean (SEM). Each experiment was designed to include at least three biological replicates, ensuring the robustness and reliability of the findings. Statistical significance is represented in the figure legends, with * indicating *p* < 0.05 and ** denoting *p* < 0.01.

## Supplementary Material

TableS1.docx

FigS2.tif

FigS1.tif

## Data Availability

The data that support the findings of this study are openly available in this manuscript and supplementary files. The raw data have been deposited to Figshare (https://doi.org/10.6084/m9.figshare.29083565.v3).
