# Supplementary material for: Exogenous proline promotes serum killing of Klebsiella pneumoniae
Source: Virulence. 2025 Aug 7;16(1):2545558. doi: 10.1080/21505594.2025.2545558 (PMC12341057; doi:10.1080/21505594.2025.2545558)
Supplement: TableS1.docx [file KVIR_A_2545558_SM3867.docx]

| **Gene** | **Primer** | **Sequence (5'-3')** |
| --- | --- | --- |
| *ompA* | Forward | CACTGGCGTTTCCCCAGTAT |
|  | Reverse | TGACCGAAGCGGTAGGAAAC |
| *ompK17* | Forward | CTGGGCGTTATCGGTTCCTT |
|  | Reverse | TAACCAACACCCACTACGCC |
| *OmpK35* | Forward | TATGCGGCCGTCATGTACTC |
|  | Reverse | GGTCTGTACGTAGCCGATGG |
| *OmpK36* | Forward | TTCTTCGGTCTGGTTGACGG |
|  | Reverse | CGGTACGACCGTTGTTGGTA |
| *putA* | Forward | ACCTGTACGTCAACCGCAAT |
|  | Reverse | CGGTACAGATACAGCGGACC |
| *proC* | Forward | GTCGCTGGTGGTCTCTATCG |
|  | Reverse | AACATCTTCGCTGCTCACCA |
| *gdhA* | Forward | GCGCTGATGACCGAACTCTA |
|  | Reverse | GCTGTTGTTGGAGAGCTTGC |

**Supplementary Table 1. List of genes and primers used in this study**
