# Supplementary figures and images for: Exogenous proline promotes serum killing of Klebsiella pneumoniae
Source: Virulence. 2025 Aug 7;16(1):2545558. doi: 10.1080/21505594.2025.2545558 (PMC12341057; doi:10.1080/21505594.2025.2545558)

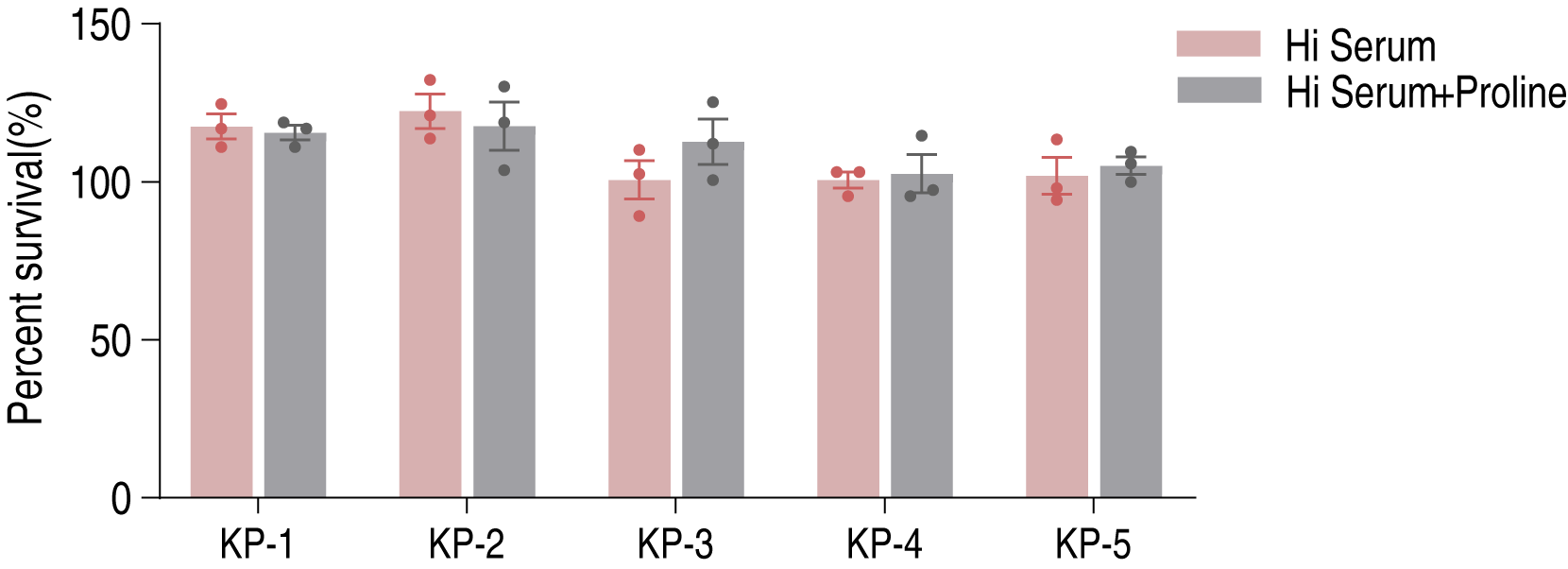

Supplement: FigS2.tif [file KVIR_A_2545558_SM3866.tif]

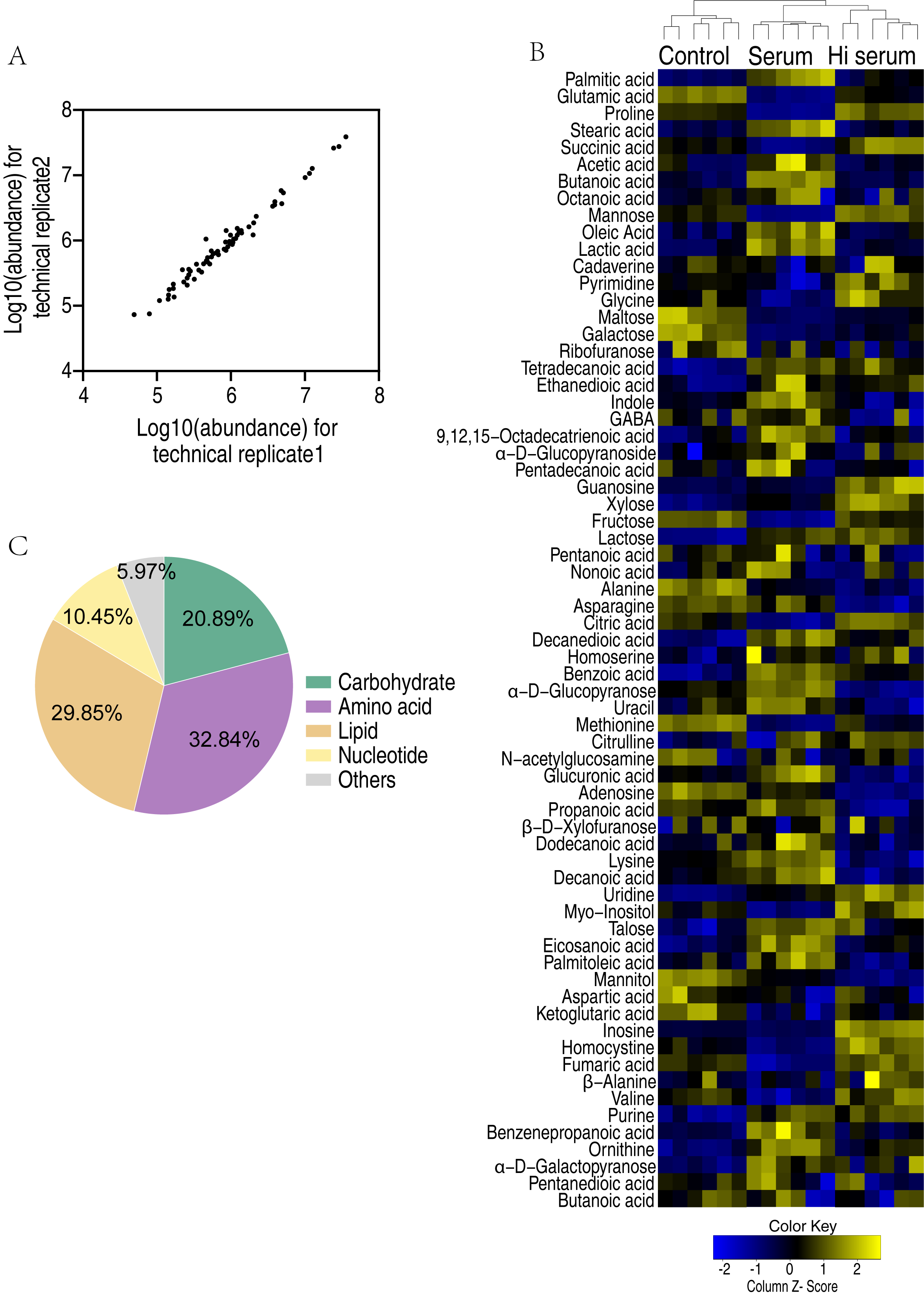

Supplement: FigS1.tif [file KVIR_A_2545558_SM3865.tif]
